# Uncovering a New Family Cluster of Gaucher Disease: A Case Report

**DOI:** 10.7759/cureus.51604

**Published:** 2024-01-03

**Authors:** Ana Carvoeiro, Miguel Costa, Joana Silva, Paula Felgueiras, Diana Guerra

**Affiliations:** 1 Internal Medicine, Unidade Local de Saúde do Alto Minho, Viana do Castelo, PRT; 2 Internal Medicine, Hospital Viana do Castelo, Viana Do Castelo, PRT; 3 Internal Medicine, Unidade Local de Saúde do Alto Minho, Hospital Conde de Bertiandos, Ponte de Lima, PRT

**Keywords:** enzyme replacement therapy, cytopenias, hepatosplenomegaly, deficiency in glucocerebrosidase, lysosomal storage disorder, gaucher disease

## Abstract

Gaucher disease (GD) is a recessive autosomal lysosomal storage disorder caused by a deficiency in glucocerebrosidase, leading to the accumulation of undigested glycolipids in the lysosomes of monocytes and macrophages. Patients with GD exhibit a spectrum of phenotypic heterogeneity and are broadly classified into three subtypes. Type 1 is the most common and is not associated with neurological damage, while types 2 and 3 are more severe, presenting with acute neuropathic and subacute neuropathic symptoms, respectively. A thorough accurate initial multisystemic assessment is crucial for evaluating the damage to all potentially affected organs and determining the disease burden. This case report highlights the intricacies of GD type 1 by providing a thorough exploration of the clinical presentation and showcasing valuable insights into the unique manifestations of the disease. The key feature was his individual and family medical history, which allowed the identification and treatment of another case within the community.

## Introduction

First described in 1882, Gaucher disease (GD) is a recessive autosomal lysosomal storage disorder caused by a deficiency in glucocerebrosidase, leading to the accumulation of undigested glucocerebroside in the lysosomes of monocytes and macrophages [1]. Gaucher cells, the affected leukocytes, tend to accumulate primarily in the bone marrow, liver, spleen, and lungs, resulting in hepatosplenomegaly and cytopenias. Moreover, GD frequently encompasses neurological and skeletal manifestations, which are the most disabling features [2,3].

The most prevalent mutations associated with this disease are N370S (p.Asn409Ser) and L444P (p.Leu483Pro) [4,5]. Patients with GD exhibit a spectrum of phenotypic heterogeneity and are broadly classified into three subtypes. Type 1 is the most common and it is not associated with acute/subacute neurological damage, while types 2 and 3 are more severe, presenting with acute neuropathic and subacute neuropathic symptoms, respectively [6]. Type 1 GD represents 90-95% of all cases of GD and, although not life-threatening, it diminishes the quality of life and increases morbidity [6,7]. Although it is a panethnic disorder, type 1 GD is especially prevalent among individuals of Ashkenazi Jewish descent [8]. In the last years, it has been reported that type 1 GD is associated with an increased risk of certain malignancies. Despite being considered a non-neuropathic type, in recent years, it has been recognized that type 1 GD may be associated with parkinsonism [9].

The median age of diagnosis typically falls between 10 and 20 years, requiring the measurement of glucocerebrosidase activity in peripheral leukocytes and molecular analysis [10]. A throughout accurate initial multisystemic assessment is crucial for evaluating the damage to all potentially affected organs and determining the disease burden.

This compelling case report highlights the intricacies of GD type 1 through a comprehensive analysis of a 40-year-old male patient. The report provides a throughout exploration of the clinical presentation, offering valuable insights into the unique manifestations of the disease. A distinctive aspect of this report lies in its emphasis on genetic and familial considerations, adding a critical dimension to the understanding of the disease's broader impact. 

## Case presentation

A 40-year-old Caucasian man presented to the emergency department with abdominal pain in the left lower quadrant lasting for one week, characterized by moderate intensity and only slight relief with paracetamol use every eight hours. The patient also reported abdominal distention, fatigue, and significant weight loss (eight kilograms in the past month, accounting for 14% of his normal body weight).

His past medical records revealed moderate asymptomatic thrombocytopenia dating back to 2010 and never studied, with a recorded minimum platelet count of 78,000. He is a former smoker with a history of 10 pack-years; he also had a well-documented family predisposition to unexplained thrombocytopenia. Multiple family members reported experiencing low platelet levels from a young age. Notably, his sister was recently diagnosed with moderate thrombocytopenia (platelets=68,000x10^12/L) during her hospital admission for the birth of her healthy child. 

During the physical examination, conjunctival pallor (+), hepatomegaly (5 cm below the right costal margin), and non-tender massive splenomegaly (10 cm below the left costal margin) were detected.

Laboratory results revealed pancytopenia (hemoglobin=11.2g/dL, white blood cells=3.10x10^9/L, and platelets=34,000x10^12/L), an erythrocyte sedimentation rate (ESR) of 26 mm/hour, a serum C reactive protein of 3 mg/dL, and a procalcitonin of 0.05 ng/mL. Liver enzymes, serum proteins, albumin levels, kidney function tests, and urine analysis were unremarkable. Coagulation tests showed a prothrombin time (PT) of 13.4s (control 11.4s), an International Normalized Ratio (INR) of 1.17, and a partial thromboplastin time (PTT) of 33.8s (control 30.0s) (Table 1). Peripheral blood smear exhibited moderate anisocytosis without appreciable morphological changes or platelet aggregates. Tests for HIV, hepatitis B, and hepatitis C were negative.

**Table 1:** Laboratory results revealing pancytopenia. Activity of glucocerebrosidase is below the reference range. The results also show a significant increase in the enzymatic activity of plasma β-D chitotriosidase and glucosylsphingosine (lyso-Gb1). LDL: Low-density lipoprotein; HDL: high-density lipoprotein

Parameter	Result	Reference Range
Hemoglobin	11.2 g/dL	13.2 - 17.2
White blood cells	3.10 x10^9/L	4.0 - 10
Platelets	34,000 x10^12/L	150 - 400
Erythrocyte sedimentation rate (ESR)	26 mm/hour	2 - 8
Serum C reactive protein (CRP)	3 mg/dL	0.3 – 1.0
Procalcitonin	0.05 ng/mL	<0.01
Prothrombin time (PT )	13.8 sec	9.7 - 13.0
Partial thromboplastin time (PTT)	34.2 sec	27.1 - 33.6
INR	1.17	
Total cholesterol	200 mg/dL	<200
LDL cholesterol	117 mg/dL	<100
HDL cholesterol	43 mg/dL	>60
Glucocerebrosidade	1.0 nmol/mg	2.8 – 19
β-D-chitotriosidase	12267 nmol/h/ml	10 - 85
Glucosylsphingosine (lyso-Gb1),	280 nmol/L	0-3.5

**Figure 1:**
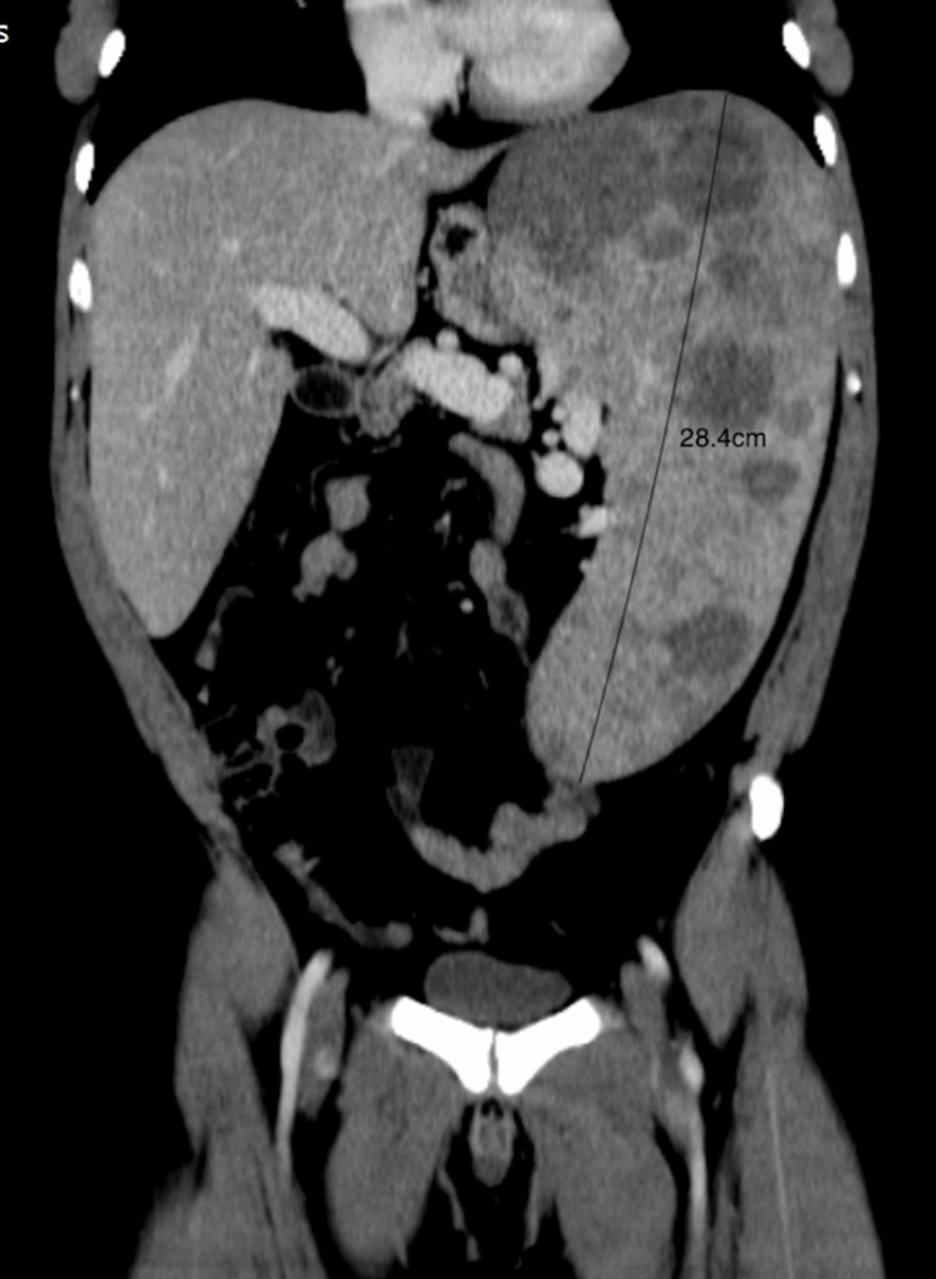
Abdominal and pelvic CT revealing significant hepatosplenomegaly and multiple hypoenhancing nodules scattered throughout the spleen.

Abdominal ultrasound revealed significant enlargement of the portal vein, marked hepatomegaly, and massive splenomegaly with multiple hypoenhancing nodules scattered throughout the spleen lacking specificity. Subsequent abdominal/pelvic CT and MRI confirmed these findings, with the liver measuring 22 cm in the midclavicular line and the spleen 28.4 cm along the bipolar axis, excluding signs of splenic vein thrombosis (Figure 1).

Given the identified hepatomegaly, massive splenomegaly, and pancytopenia, a myelogram and bone marrow biopsy were performed. Considering the patient's long-standing, non-studied thrombocytopenia, differential diagnosis included storage disease, hematological disorders (such as leukemia/lymphoma), and autoimmune diseases. A screening technique for storage diseases, the dried blood spot (DBS), was also conducted.

**Figure 2:**
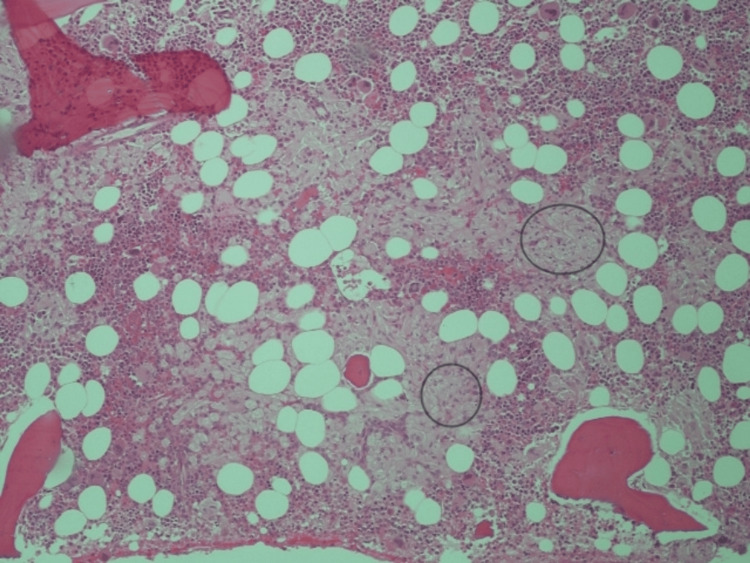
Bone marrow biopsy revealing diffuse infiltration by Gaucher cells (some of them delimited by circles).

**Figure 3:**
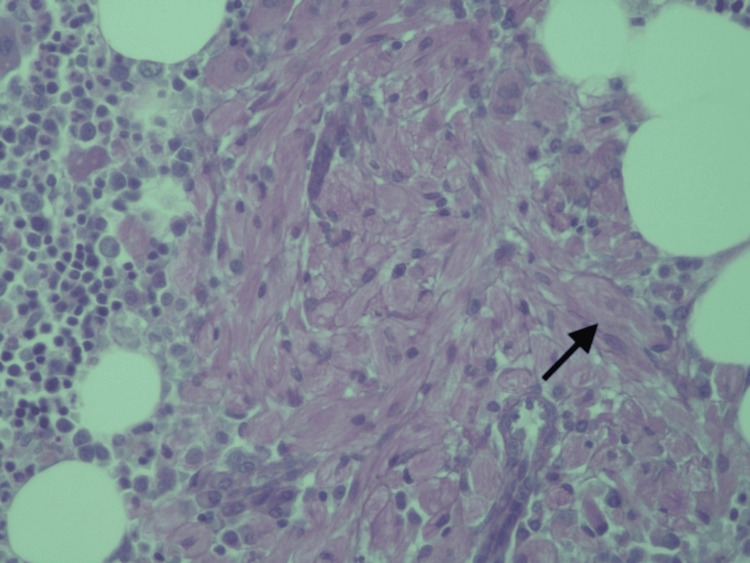
Bone marrow histology showing macrophages laden with glucocerebroside (Gaucher cell, arrow). Periodic acid-Schiff (PAS) and periodic acid–Schiff–diastase (PASD) staining are positive in Gaucher cells (diastase resistant), which is translated by strongly positive granular or fibrillar material in the cytoplasm, with the characteristic 'wrinkled tissue paper' appearance.

**Figure 4:**
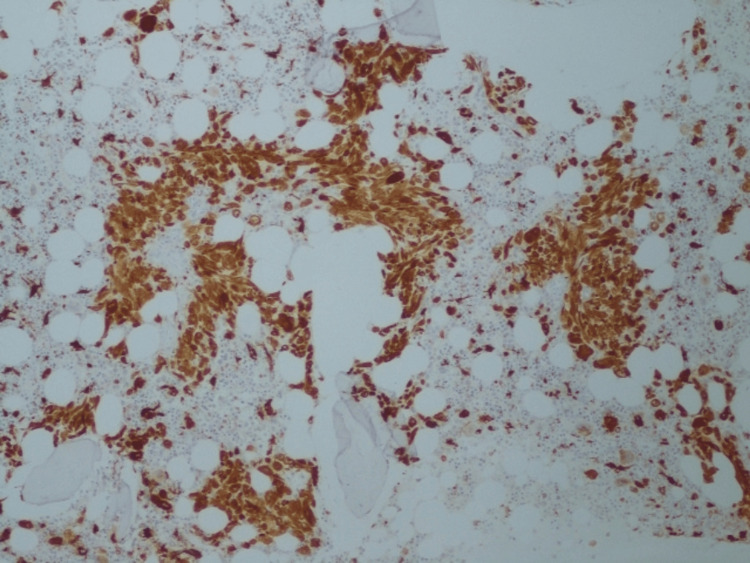
Immunohistochemical stain for CD68 helped identify isolated Gaucher's cells, which are hystiocytic in nature.

The bone marrow aspirate showed no signs of hematological disease, but the biopsy indicated morphological and immunohistochemical characteristics suggestive of GD Type 1 (Figures 2-4). DBS results further supported the GD diagnosis, showing a partial deficit in β-glucosidase activity and a significant increase in the enzymatic activity of β-D-chitotriosidase, a marker used for treatment effectiveness and prognostic evaluation. Additionally, elevated levels of glucosylsphingosine (lyso-Gb1), a reliable biomarker, were detected (Table 1).

GD was confirmed through leukocyte glucocerebrosidase enzyme activity assessment (1.0 nmol/hr/mg) (Table 1) and genetic analysis, revealing compound heterozygosity for N370S (p.Asn409Ser) and L44P (p.Leu483Pro) mutations. These comprehensive findings contribute significantly to the understanding of GD Type 1 in this patient, providing valuable insights for both clinical management and further research endeavors.

While there is no cure for GD, enzyme replacement therapy with imiglucerase was initiated, leading to a definitive diagnosis. Afterward, another GD case within the patient's family was discovered: the patient's sister, previously diagnosed with thrombocytopenia of 68 000 platelets, had also a DBS showing partial enzyme deficit and she is currently being followed up at the same reference center where our patient is followed. This case not only emphasizes the importance of early diagnosis and treatment but also emphasizes the significance of detailed past and family medical history in identifying potential cases of rare diseases.

## Discussion

GD is a rare autosomal recessive genetic disorder caused by a deficiency of the lysosomal enzyme glucocerebrosidase/β-glucosidase, resulting in the accumulation of glucocerebroside in lysosomes of macrophages [1]. This disorder manifests with a broad spectrum of clinical presentations, including cytopenia, hepatosplenomegaly, skeletal manifestations, and, in some cases, neurological impairment, the latter being a particularly debilitating feature.

GD is the most common lysosomal storage disease worldwide, with an estimated frequency of 1 in 40,000 individuals [2]. In Portugal, around 140 individuals are estimated to have GD being the birth prevalence in the Portuguese population of 1.4 per 100 000 live births [11-13].

While this disorder can affect people of all races and ethnic backgrounds and may manifest at any age, a significant number of cases are typically diagnosed during adulthood [14]. Despite symptoms often emerging in the early twenties, the diagnosis of rare diseases like GD is frequently delayed [15]. One in six patients with GD may wait up to seven years before receiving a definitive diagnosis, primarily due to the challenge posed by other diseases with similar presentations [13].

Three major phenotypic presentations of GD have been identified: Type 1, the most reported worldwide, does not exhibit associated neurological symptoms, while types 2 and 3 are characterized by neurological impairment. Type 1, considered "the adult type," is marked by painless hepatosplenomegaly, leading to significant abdominal distension and cytopenias, often resulting from hypersplenism and bone marrow infiltration by Gaucher cells [4,16]. Notably, some patients with type 1 may only present with splenomegaly [6,7].

Our patient, exhibiting massive splenomegaly, prompted a thorough investigation, given the limited number of conditions associated with such a presentation. Considering the patient's extreme weight loss and to exclude hematologic malignancy, a bone marrow examination was performed.

The personal and family history of thrombocytopenia were key indicators, raising a high degree of diagnostic suspicion for a storage disorder. Nowadays, diagnosing lysosomal storage diseases is enabled by painless techniques such as DBS on filter paper [17], as applied in this case.

After confirming a partial deficit in glucocerebrosidase activity through DBS, subsequent leukocyte assessment and genetic analysis identified two mutations, confirming GD. Additionally, biomarkers such as chitotriosidase and lyso-Gb1 [18-20] were measured for disease monitoring.

## Conclusions

The authors highlight the need for prompt consideration of lysosomal storage diseases in cases of unexplained massive splenomegaly, as early detection significantly impacts patient management and prognosis. This case report contributes to the scientific community's understanding of a rarely suspected diagnostic possibility, emphasizing the importance of individual and family medical history in identifying and treating other cases within a community.
